# Draft Genome Sequence of *Lactobacillus pasteurii* CRBIP 24.76^T^

**DOI:** 10.1128/genomeA.00660-13

**Published:** 2013-08-22

**Authors:** Sylvie Cousin, Dominique Clermont, Sophie Creno, Laurence Ma, Valentin Loux, Chantal Bizet, Christiane Bouchier

**Affiliations:** Institut Pasteur, Centre de Ressources Biologiques de l’Institut Pasteur, Département de Microbiologie, Paris, Francea; Institut Pasteur, Plate-forme Génomique, Département Génomes et Génétique, Paris, Franceb; INRA, UR1077 Mathématique, Informatique et Génome, Jouy-en-Josas, Francec

## Abstract

We report the draft genome sequence of the type strain *Lactobacillus pasteurii* CRBIP 24.76, which is closely related to *L. gigeriorum* CRBIP 24.85^T^, isolated from a chicken crop. The total length of the 29 contigs is about 1.9 Mb, with a G+C content of 40% and 1,946 coding sequences.

## GENOME ANNOUNCEMENT

The genus *Lactobacillus* represents the largest group of facultatively anaerobic, catalase-negative, non-spore-forming, Gram-positive, rod-shaped organisms which produce lactic acid as a major end product of metabolism (1); the genus currently contains >150 recognized species or subspecies. Species of the genus *Lactobacillus* have been isolated from a wide range of habitats, such as the oral cavity, vagina, and gastrointestinal tracts of humans and animals, food, vegetation, and sewage, and are of great commercial importance due to their use in the production of a range of fermented dairy, meat, and vegetable products. *Lactobacillus* species are also the most widely used probiotics, aimed at promoting a healthy lifestyle. *Lactobacillus pasteurii* strain 1517^T^ was isolated in the early 1960s and deposited in the Collection de l’Institut Pasteur under the strain name CRBIP 24.76^T^. Unfortunately, according to the depositor archives, the origin of the strain is not known. This strain represents a new species (2) that is well discriminated from its closest relative *Lactobacillus gigeriorum* CRBIP 24.85^T^, isolated from a chicken crop (3).

Here, we report the draft genome sequence of *L. pasteurii* CRBIP 24.76^T^, which was obtained using a whole-genome strategy based on Illumina paired-end sequencing with an insert length of 420 bp (Illumina genome analyzer HiSeq 2000). Quality-filtered reads (16,681,432 reads, 89 bases mean read length, ~730-fold coverage) were assembled using ABySS (version 1.2.6 [4]) with a k-mer of 40 bases. All reads were assembled into 30 contigs. The total length of the 30 contigs is 1,905,399 nucleotides (nt), with a G+C content of 40%. The length of the longest contig is 313,402 bases, and the smallest is 1,359 bases. The contigs were annotated with the AGMIAL platform (5), an integrated bacterial genome annotation system. The prediction of coding sequences used the self-training gene detection software SHOW based on hidden Markov models (http://genome.jouy.inra.fr/ssb/SHOW/). tRNAs and rRNAs were detected using tRNAscan-SE (6) and RNAmmer (7) softwares, respectively. The predicted conserved domain sites were 1,946 (1,917 complete), among which 11 rRNA operons with 3, 3, and 5 copies of 23S, 5S, and 16S, respectively, were detected, as well as 52 tRNA genes.

### Nucleotide sequence accession numbers. 

The strain is publicly available in two European Biological Resource Centres under the no. CRBIP 24.76^T^ and DSM 23907^T^. The draft of the whole-genome sequencing project has been deposited in EMBL under the accession no. CAKD01000001 to CAKD01000030. The version described in this paper is the first version. 
